# Hospital Surge Capacity Preparedness in Disasters and Emergencies: Protocol for a Systematic Review

**DOI:** 10.3390/ijerph192013437

**Published:** 2022-10-18

**Authors:** Md. Khalid Hasan, Sarker Mohammad Nasrullah, Annalisa Quattrocchi, Pedro Arcos González, Rafael Castro Delgado

**Affiliations:** 1 Institute of Disaster Management and Vulnerability Studies, University of Dhaka, Dhaka 1000, Bangladesh; 2 Unit for Research in Emergency and Disaster, Faculty of Medicine and Health Sciences, University of Oviedo, 33006 Oviedo, Spain; 3 Department of Primary Care and Population Health, Medical School, University of Nicosia, Nicosia 2408, Cyprus

**Keywords:** hospital, surge capacity, preparedness, disaster, emergency, medical surge, systematic review

## Abstract

Hospitals’ medical surge preparedness or surge capacity preparedness plays a significant role in reducing mortalities and in the treatment of severe injuries in disasters and emergencies. Though actions or activities for surge capacity preparedness of hospitals are discussed in several studies, they remain fragmented and need to be compiled. This systematic review will provide a comprehensive synthesis of evidence of actions or steps taken to strengthen hospitals’ medical surge preparedness in disasters and emergencies, which will eventually help develop surge capacity programs and relevant policies. All the studies published in peer-reviewed journals between 1 January 2016 and 30 July 2022, with full text available, will be included in this review. Seven electronic databases—PubMed, Scopus, MEDLINE, CINAHL, Embase, PsycINFO, and Ovid—will be searched. Two reviewers will independently screen the titles and abstracts using the eligibility criteria, review full-text articles, and extract data with the help of CADIMA software. A third reviewer will help resolve any discrepancies during the whole process. The extracted data will be narratively synthesized with the key characteristics and findings of the studies. The NIH quality assessment tools will be used to scale up the the quality of the retrieved quantitative studies. Moreover, the mixed methods appraisal tool (MMAT) and Noyes et al. guidelines will be used to assess the mixed methods studies and qualitative studies quality assessment, respectively.

## 1. Background

Large-scale disasters, both natural and human-made, and emergencies can cause the severe destruction of lives and livelihoods by creating serious disorders in the affected people’s social, economic, and organizational activities, including the health care system [1,2,3,4]. Although health care systems should play a crucial role in reducing morbidity and mortality during disasters and emergencies, many hospitals might not be well prepared to face these challenges due to inadequate medical surge preparedness or simply the demand for health care superseding the hospital surge capacity [3,4,5]. Therefore, medical surge preparedness should be an integral and essential part of hospital preparedness as well as health system programs, especially in disaster-prone countries, to cope with disasters and emergencies by reducing the losses of lives and disabilities [3,4,5,6].

A medical surge occurs when the needs of clinical and/or patient volumes exceed the hospital’s service limits [7]. During a mass casualty incidence due to disasters or emergencies, the hospital’s emergency departments (EDs) become the epicenter of healthcare deliveries and seldom need to function above their original capacity. Due to the massive influx of patients, overcrowdedness, exacerbated EDs admissions, disrupted care flow, and constrained resources, hospitals may compromise the quality and safety of patient care during disasters and emergencies if they are not well prepared to expand their surge capacity [8].

However, there is no single definition and measurement standard of surge capacity in healthcare or disaster planning [4,6,9]. The American College of Emergency Physicians (ACEP) defined surge capacity as “a measurable representation of a health care system’s ability to manage a sudden or rapidly progressive influx of patients within the currently available resources at a given point in time” [10]. However, the measurable representation is neither well defined nor operational [8]. Adams [9] has given a comprehensive definition of surge capacity as “the ability to obtain adequate staff, supplies and equipment, structures and systems to provide sufficient care to meet immediate needs of an influx of patients following a large-scale incident or disaster”. This definition reflects the theoretical constructs of the four domains or components of hospital surge capacity: (1) staff or human resources; (2) stuff or equipment and supplies; (3) structure or physical space; and (4) systems that include integrated management policies and processes [8,9,11,12], which were initially proposed by Kelen and McCarthy [13] in their seminal work, titled “The science of Surge”. Therefore, before developing and implementing programs for hospitals’ surge capacity, the primary evaluation and risk assessment of crucial components of surge capacity are essential to achieving the programs’ successes [4].

Medical surge preparedness in hospitals can improve surge capacity planning by linking preparedness actions to institutional triggers across the surge capacity continuum, and significantly reducing the injuries and deaths of patients [4,7]. Several studies discussed preparedness actions and activities to increase the surge capacity of hospitals; however, those actions change with time, depending on the resources and capacities of the hospitals [4,10,14,15,16,17,18,19]. Therefore, the hospital surge capacity program must be revised and updated frequently. Moreover, a systematic identification, synthesis, and appraisal of the findings of literature related to the surge capacity preparedness of hospitals will provide a comprehensive understanding of the mechanisms of improving and strengthening the surge capacity of hospitals, and help to develop surge capacity-related programs and policies. A previous review of the surge capacity of hospitals with a preparedness approach was conducted by Sheikhbardsiri et al. [4], who synthesized the literature from 2001 to 2015, which is quite outdated. Thus, this study will attempt to fill the gaps by systematically reviewing and synthesizing the recent evidence on the surge capacity preparedness of hospitals in disasters and emergencies.

### Research Objectives and Questions

The overall objective of this systematic review is to identify, integrate, and synthesize the available evidence on the surge capacity preparedness of hospitals in disasters and emergencies. The review will try to answer the following questions:

- What preparedness activities or actions improve/increase the medical surge capacity of hospitals in disasters and emergencies?
- Are there any differences between private and public hospitals’ medical surge preparedness in disasters and emergencies? If any, what are the differences?
- Does the medical surge capacity of hospitals differ by location or region? If any, then how much difference is there? and
- Which are the barriers to the medical surge capacity of hospitals in disasters and emergencies?

## 2. Methods

### 2.1. Study Design

This systematic review protocol is informed by the preferred reporting items for systematic reviews and meta-analysis protocols (PRISMA-P) (Supplemental Materials, Table S1). Narrative synthesis methods [20] and the PRISMA 2020 statement [21] will be used to ensure the complete transparency and completeness of the reporting, mainly related to the methods used to identify, select, appraise, and synthesize the included studies. The final report will account for any amendments to the protocol and rationale during the systematic review. The protocol will be registered with the PROSPERO database of systematic reviews (PROSPERO registration number: CRD42022360332).

### 2.2. Ethical Considerations

The present study will be based solely on previously reported studies. Hence, there is no concern that requires ethical approval.

### 2.3. Patient and Public Involvement

No patients nor members of the public are involved in conducting the review.

### 2.4. Eligibility Criteria

The first inclusion criterion of the systematic review is the peer-reviewed full-text articles published in English that investigated the surge capacity or medical surge preparedness of private and public hospitals in disasters and emergencies. The second inclusion criterion is that the literature must consider at least one component (staff, stuff/supply, system, and structure/space) of medical surge capacity in a disaster. Moreover, qualitative, quantitative, and mixed methods research designs will be incorporated, and no restrictions will be placed regarding the geographical location of the studies.

Since the previous study on hospital surge capacity synthesized evidence from 2000 to 2015 [4], the studies conducted before 2016 and those investigating the medical surge capacity/preparedness of other treatment locations or places, except hospitals, will be excluded from the study. However, selection bias might exist in this systematic review in terms of publication bias because grey literature will be excluded from the review (Table 1).

### 2.5. Information Sources

Peer-reviewed articles published from 1 January 2016 to 30 July 2022 will be retrieved using the following databases: PubMed, Scopus, MEDLINE, CINAHL, Embase, PsycINFO, and Ovid. These databases usually index all the published articles related to hospital surge capacity and preparedness. Moreover, previous systematic reviews related to surge capacity or preparedness also used these databases for literature searching. In addition, to ensure literature saturation, the reference lists of all the eligible studies found in the database search will be screened to identify possible additional relevant articles.

### 2.6. Search Strategy

A search strategy will be developed on PubMed and adopted in other databases using MESH terms and keywords, as shown in Table 2. The search will apply Boolean operators (‘AND’, ‘OR’, and ‘NOT’) and truncations (*), depending on the databases’ specifications. The search terms will be searched for in the title, abstract, and topic fields or all fields if the databases allow such delimiters. The search strategy will be finalized after a consultation with a research librarian.

### 2.7. Study Records

#### 2.7.1. Data Management

All the search results from the databases mentioned above will be extracted as RIS files and exported to CADIMA—an open-access online tool for supporting and reporting systematic review and meta-analysis—to remove duplicate articles and complete the screening process.

#### 2.7.2. Selection Process

According to the eligibility criteria, two independent reviewers (MKH and SMN) will scan the titles and abstracts of all retrieved articles from the database searches using the CADIMA software. After the screening, the results will be compared and if any disharmonies appear, a third reviewer (PAG/RCD) will resolve the issues. Then, the potentially eligible articles of this stage will proceed to full text-screening (next step) by two independent reviewers (MKH and SMN), and the reason for exclusion will be recorded; and any disagreement between them will be resolved with the help of a third reviewer (PAG/AQ/RCD). A PRISMA 2020 flow diagram will be used to represent the review study selection process.

#### 2.7.3. Data Collection Process

A standardized data collection template (Appendix A, Table A1) will be developed in the CADIMA software, along with a detailed instruction manual that will be used to collect specific information from the eligible full-text articles by two independent reviewers (MKH and SMN). In the case of missing details of the articles, reviewers will email the corresponding authors to request the information.

### 2.8. Data Items

The extracted information will be based on the PICO (population, intervention, comparison, and outcome) structure [22] and will include the following:

- participant characteristics: e.g., hospital location, types of hospital, location of hospitals (urban or rural, country), no. of hospitals, hospital size, hospital beds, built environment, and types of surge capacity component;
- exposure to disaster or emergency: e.g., type of disasters, details of the disaster, and type of emergencies;
- primary outcomes: e.g., identifying ways of improving medical surge preparedness/capacity in disasters and emergencies; and
- secondary outcomes: e.g., identifying barriers to hospital surge capacity/preparedness in disasters and emergencies.

General information (e.g., author, article title, reviewer, date of data extraction, record number), study characteristics (e.g., study design, study aim), missing data, and quality assessments will also be collected about the full-text eligible articles. After the data extraction, the reviewers will compare their results and if any disagreement appears, a third reviewer will dissolve the issue.

### 2.9. Risk of Bias Individual Studies/Quality Assessment

Two reviewers will assess the quality and risk of bias in all the included studies using the NIH quality assessment tools [23]. As we expected, various types of studies, such as cross-sectional, RCTs, qualitative, and others will be included in the review; therefore, a comprehensive, practical, and reliable appraisal tool like the NIH tools will be suitable for the quality checking of quantitative types of studies [24]. The studies’ quality will be rated as good, fair, and poor using NIH tools. We will use the risk of bias assessment results to assess the confidence of the results and the limitations of studies. Moreover, Noyes et al. [25] guidance will be followed for qualitative studies, and the mixed methods appraisal Tool (MMAT) [26] will be used to assess the quality of mixed methods studies. Any study with serious methodological concerns will be excluded. In the case of any disagreement between the results of the two reviewers, a third reviewer will resolve the issues.

### 2.10. Data Synthesis

Since the included studies are expected to be heterogeneous in methods, measurements, and results, the extracted data will be narratively synthesized with the key characteristics and findings of the studies. One of the advantages of narrative synthesis is that it can provide detailed responses to the questions directing the review [27]. The narrative synthesis will provide detailed descriptions of: (1) concepts on hospital surge capacity preparedness or medial surge preparedness; (2) time to initiate preparedness activities for surge capacity; (3) surge preparedness activities; and (4) barriers to medical surge preparedness/surge capacity. A summary table of the studies’ characteristics will also be presented with their methodological quality assessment.

### 2.11. Meta-Bias & Confidence in Cumulative Evidence

Meta-bias occurs due to publication bias, the statistically significant results being more likely to be published than non-significant results; and reporting bias, the selective reporting of outcomes for their direction, magnitude, and significance [21]. For confidence in cumulative evidence, the GRADE methodology is usually applied and performed for RCTs or well-designed cohort studies, which is not the case in this systematic review. Though the eligible studies will be primarily non-RCTs, cross-sectional, mixed-methods, or qualitative, we do not plan any assessment of meta-bias or confidence in cumulative evidence.

## 3. Discussion

Healthcare facilities should have a dedicated plan and preparedness for surge capacity, so that they can quickly expand their accommodation for additional patients and give quality care services to those emergency healthcare seekers [4,7,28]. Adequate medical surge preparedness can also effectively reduce the severity of injuries and losses of life in an emergency or disaster. By far, very few protocols, systematic reviews, and meta-analyses have been published on the medical surge preparedness of hospitals in disasters and emergencies. We found only one systematic review that reviewed hospital surge capacity from a preparedness perspective, which is now outdated. Therefore, a new systematic review with recent evidence is required to comprehensively identify and synthesize the preparedness actions and activities for increasing hospital surge capacity. Additionally, it is anticipated that this study will provide vital information for healthcare managers, public health stakeholders, researchers, and policymakers to develop and design surge capacity programs for hospitals and other healthcare facilities.

## 4. Conclusions

The findings of this systematic review will provide comprehensive knowledge about hospital surge capacity dimension-wise measures and actions that will help to increase the surge capacity preparedness of hospitals in disasters and emergencies. The study findings will also be helpful for all types of public and private hospitals, regardless of geographical location; i.e., developed countries, developing countries, and underdeveloped countries.

### Strengths and Limitations of This Study

- This study will strictly adhere to the preferred reporting items for systematic review and meta-analysis (PRISMA);
- The selected articles will be critically assessed for their risk of bias using the NIH quality assessment tools, mixed methods appraisal tool (MMAT), and Noyes et al. guidelines;
- This systematic review will synthesize the updated comprehensive evidence on the medical surge preparedness of hospitals, irrespective of location or geography;
- As this systematic review will include only English articles, potential studies in other languages may be missed; and
- As grey literature will not be searched, there is a possible chance of publication bias.

## Figures and Tables

**Table 1:** Inclusion and exclusion criteria for the study.

	Inclusion Criteria	Exclusion Criteria
**Population**	Hospitals	Other healthcare facilities, except for hospitals
**Intervention**	Actions or activities for hospital surge capacity preparedness in disasters and emergencies	Routine activities in normal time
**Comparison**	None	
**Outcome**	Measures of hospital surge capacity preparedness in disasters and emergencies	
**Study**	All quantitative, qualitative, and mixed methods peer-reviewed studies, including RCTs, non-RCTs, cohort studies, cross-sectional studies, and surveys.The study must consider at least one component (staff, stuff/supply, system, and structure/space) of medical surge capacity	Protocols, editorials, letters to editors, commentaries, conference abstracts and posters, and opinion pieces that are not peer-reviewed, including grey literature
**Settings**	Irrespective of location and geography	
**Date Range**	1 January 2016 to 30 July 2022	Published prior to January 2016
**Language**	English	Studies published in languages other than English

**Table 2:** Search strategy piloted in PubMed, PsycINFO, and Scopus.

SI	Database	Search Terms/Query
1	PubMed	((((surge OR “surge capacity”)) AND (“hospital*” OR “health care” OR “health centre*” OR “health center*” OR “health facilit*” OR “hospital emergency department”)) AND (capacity OR capacities OR preparedness OR preparation OR action*)) AND (“natural hazard*” OR earthquake* OR flood* OR storm* OR cyclone* OR drought* OR hurricane* OR tsunam* OR tornado* OR fire* OR wildfir* OR emergency OR emergenc* OR crisis OR “natural disaster*” OR disaster* OR hazard* OR tragedy OR catastrophe OR “mass casualty incident”)
2	PsycINFO	(surge or surge capacity) AND (hospital* or “health care” or “health centre*” or “health center*” or “health facilit*” or “hospital emergency department*”) AND (capacity or capacities or preparedness or preparation or action*) AND (“natural hazard*” or earthquake* or flood* or storm* or cyclone* or drought* or hurricane* or tsunam* or tornado* or fire* or wildfir* or emergency or emergenc* or crisis or “natural disaster*” or disaster* or hazard* or tragedy or catastrophe or “mass casualty incident*”)
3	Scopus	TITLE-ABS-KEY ((((( surge OR “surge capacity”)) AND (“hospital*” OR “health care” OR “health centre*” OR “health center*” OR “health facilit*” OR “hospital emergency department” )) AND (capacity OR capacities OR preparedness OR preparation OR action*)) AND (“natural hazard*” OR earthquake* OR flood* OR storm* OR cyclone* OR drought* OR hurricane* OR tsunam* OR tornado* OR fire* OR wildfir* OR emergency OR emergenc* OR crisis OR “natural disaster*” OR disaster* OR hazard* OR tragedy OR catastrophe OR “mass casualty incident”))

## Supplementary Materials

The following supporting information can be downloaded at: https://www.mdpi.com/article/10.3390/ijerph192013437/s1, Table S1: Reporting checklist for protocol of a systematic review and meta-analysis (PRISMA-P Statement—Checklist of items).

## Appendix A

**Table A1:** Example of the data extraction template.

Reviewer:		Date:
Author:		Year:
Journal:		Record number:
Article title:		
**Study description**		**Page number**
Objectives:		
Methods:		
Settings/sampling:		
No. of hospitals:		
Country(s):		
Location of hospital(s) (rural/urban):		
Type of hospital surge capacity components:		
Data analysis:		
Study limitations:		
Author’s conclusion:		
Additional comments:		
**Findings**		**Page number**
Main concept:		
Definition of surge capacity:		
Surge preparedness activities:		
Barriers to surge preparedness:		
Time to initiate surge capacity:		
**Evidence**	Unequivocal □	Credible □ Unsupported □
**Data extraction completed:**	Yes □	No □
