# Supplementary material for: Hospital Surge Capacity Preparedness in Disasters and Emergencies: Protocol for a Systematic Review
Source: Int J Environ Res Public Health. 2022 Oct 18;19(20):13437. doi: 10.3390/ijerph192013437 (PMC9603163; doi:10.3390/ijerph192013437)
Supplement: Supplementary file 1 [file ijerph-19-13437-s001.zip › ijerph-1977923-supplementary.pdf]

Supplemental Material

Table S1: Reporting checklist for protocol of a systematic review and meta-analysis (PRISMA-P Statement—Checklist of items)

Section and topic	Item No	Page number of the manuscript
**ADMINISTRATIVE INFORMATION**		
Title:		
Identification	1a	1
Update	1b	2
Registration	2	7
Authors:		
Contact	3a	1
Contributions	3b	7
Amendments	4	N/A
Support:		
Sources	5a	7
Sponsor	5b	7
Role of sponsor or funder	5c	7
**INTRODUCTION**		
Rationale	6	2
Objectives	7	2
**METHODS**		
Eligibility criteria	8	3-4
Information sources	9	4
Search strategy	10	4-5
Study records:		
Data management	11a	5
Selection process	11b	5
Data collection process	11c	5
Data items	12	5
Outcomes and prioritization	13	5
Risk of bias in individual studies	14	5-6
Data synthesis	15a -15d	6
Meta-bias(es)	16	6
Confidence in cumulative evidence	17	6
